# Blood-Based Epigenetic Aging Signatures in D3GHR Carriers: An Exploratory Pilot Study of Metabolic Adaptation and Aging-Related Pathways

**DOI:** 10.3390/ijms27125181

**Published:** 2026-06-08

**Authors:** Ori Berger, Maayan Insler, Ghadeer Falah, Gil Ben David, Lital Sharvit, Shmuel Springer, Ran Talisman, Gil Atzmon

**Affiliations:** 1 Plastic Surgery Department, Barzilai University Medical Center, Ashkelon 7830604, Israel; 2 Department of Human Biology, Faculty of Natural Sciences, Medical School, University of Haifa, Haifa 3498838, Israelgili.bd@gmail.com (G.B.D.);; 3 The Neuromuscular & Human Performance Laboratory, Department of Physical Therapy, Faculty of Health Sciences, Ariel University, Ariel 4070000, Israel; shmuels@ariel.ac.il

**Keywords:** d3GHR, DNA methylation, epigenetic clock, Horvath clock, ACAT2, CYP1A1, metabolic adaptation, longevity, growth hormone receptor, pilot study

## Abstract

The exon 3 deletion polymorphism in the growth hormone receptor gene (d3GHR) is associated with altered GH signaling and longevity-related phenotypes, yet its relationship with blood-based epigenetic aging remains unclear. We analyzed whole-blood DNA from 21 unrelated adults recruited at Laniado Medical Center to determine whether the d3GHR genotype was associated with differential DNA methylation in skin-aging-related genes and altered age acceleration across established DNA methylation clocks. Genome-wide methylation was profiled using the Infinium MethylationEPIC v2.0 array, focusing on 1098 CpG sites linked to wrinkling, pigmentation, and extracellular matrix remodeling. No significant single-CpG methylation differences were detected within the targeted panel. However, two promoter-proximal differentially methylated regions (DMRs) were identified near *CYP1A1* (FWER = 0.014) and *ACAT2* (FWER = 0.026). Notably, only the pan-tissue Horvath clock showed a significant genotype effect, with marked age acceleration in d3/d3 carriers (mean Δ ≈ +14.5 years, *p* = 0.0179) that persisted after adjustment for chronological age. In contrast, second-generation clocks such as PhenoAge showed a non-significant trend toward deceleration. These findings suggest a preliminary association between d3GHR genotype, clock-specific epigenetic age acceleration and promoter-level methylation signatures near metabolic and stress-response genes. The observed Horvath acceleration may reflect systemic metabolic or immune adaptation rather than direct structural senescence in core skin-aging gene programs in blood. Given the very small d3/d3 subgroup, these findings should be interpreted strictly as exploratory pilot observations and cannot establish reproducible genotype-specific effects without validation in larger independent cohorts.

## 1. Introduction

Skin aging is a complex, multifactorial process characterized by the progressive loss of proliferative capacity and structural integrity. Clinically, these changes manifest as wrinkles and laxity, while biologically they are driven by coordinated alterations in hormonal signaling, cellular homeostasis, and epigenetic regulation [1]. The skin is increasingly recognized not merely as a passive target of systemic signals, but as an active endocrine-responsive organ. Genetic studies further support the concept that skin-aging phenotypes reflect inherited and systemic biological variation. A genome-wide association study of facial skin youthfulness identified candidate loci near *KCND2*, *DIAPH2*, and *EDEM1*, supporting a genetic contribution to healthy skin-aging phenotypes [2]. In addition, studies of skin fluorescence, a non-invasive marker of cutaneous advanced glycation end products, have shown that genetic variation, glycation biology, and systemic metabolic factors contribute to interindividual differences in skin-based aging and tissue-damage markers [3,4]. Growth hormone receptors (GHR) are widely expressed in cutaneous tissues, including keratinocytes, fibroblasts, and melanocytes, where growth hormone (GH) signaling regulates key processes ranging from collagen synthesis to wound repair [5,6,7,8].

These repair processes are strongly influenced by epigenetic mechanisms, particularly DNA methylation. Unlike genetic mutations, which alter the underlying DNA sequence, DNA methylation modulates gene expression in a dynamic and context-dependent manner, serving as a key regulator of cellular differentiation, development, and tissue homeostasis [9]. Advances in genome-wide methylation profiling have enabled the identification of defined CpG methylation patterns that form the basis of “epigenetic clocks.” These clocks provide quantitative estimates of biological age and age acceleration and are increasingly used as molecular biomarkers of aging [9].

A key intersection between endocrine regulation and epigenetic aging is the exon-3 deletion polymorphism of the growth hormone receptor gene (d3GHR). This polymorphism produces a shorter receptor isoform that has been associated with altered GH sensitivity and longevity-related phenotypes [9]. Previous studies have suggested that d3GHR carriers may exhibit distinct methylation profiles and potentially younger epigenetic age in peripheral blood, supporting the hypothesis that GH-receptor variation may influence aging through epigenetic regulation.

Despite reported associations between d3GHR and longevity-related phenotypes, it remains unclear whether this variant is linked to epigenetic signatures relevant to skin aging. Because whole-blood methylation profiles can reflect systemic endocrine, immune, and metabolic states that may influence skin physiology, they may provide indirect insight into genotype-driven aging pathways. In this exploratory pilot study, we primarily examined whether the d3GHR genotype was associated with blood-based epigenetic aging signatures and DNA methylation age across established epigenetic clocks. As a secondary exploratory analysis, we evaluated CpG sites mapped to genes implicated in skin-aging-related biological pathways, including extracellular matrix remodeling, pigmentation, and tissue repair. Because no direct clinical or physiological measures of skin aging were available, these analyses were intended to assess systemic blood-based epigenetic signatures rather than clinical cutaneous aging phenotypes.

## 2. Results

### 2.1. Subject Characteristics and Genotyping

We analyzed 21 unrelated individuals recruited from Laniado Medical Center. Genotyping of the GHR exon 3 polymorphism identified 7 participants (33.3%) with the wild-type genotype (fl/fl), 11 participants (52.4%) who were heterozygous (fl/d3), and 3 participants (14.3%) who were homozygous for the exon 3 deletion (d3/d3). Although the absolute number of d3/d3 participants was small, the observed genotype distribution, including the lower frequency of d3/d3 homozygotes, was broadly consistent with reported GHR exon 3 genotype distributions, in which d3/d3 homozygosity is expected to represent a minority subgroup [9]. Given that the d3/d3 subgroup included only three individuals, all comparisons involving this genotype were interpreted as descriptive and exploratory, irrespective of nominal statistical significance.

Chronological age was broadly similar across genotype groups (Table 1). One-way ANOVA showed no statistically significant difference in age among groups (F(2,18) = 1.85, *p* = 0.19), consistent with the Kruskal–Wallis test (H = 2.82, *p* = 0.24). No statistically significant age difference by genotype was detected; however, given the small sample size and the higher mean age observed in the d3/d3 subgroup, residual confounding by age cannot be excluded.

Functional status appeared to vary across genotype groups. Accordingly, d3/d3 carriers showed numerically lower physical activity scores (*p* = 0.25) and lower physical performance scores than fl/fl and fl/d3 participants, although these differences were not statistically significant (physical performance score: *p* = 0.52; Table 2). Given the small number of d3/d3 carriers, these comparisons should be interpreted as descriptive.

To examine whether the d3GHR genotype was associated with epigenetic variation in molecular pathways relevant to skin aging, we analyzed a targeted panel of 1098 CpG sites mapped to genes with established roles in wrinkling, dermal elasticity, pigmentation, and extracellular matrix (ECM) remodeling. This panel was curated from genome-wide association studies and the mechanistic dermatology literature [5,6,7,8].

The targeted gene set included genes implicated in wrinkle formation, such as *IRF4*, *SLC45A2*, *SPATA33*, and *VAV3*; dermal elasticity and laxity, such as *COL1A1*, *COL1A2*, *COL13A1*, *ELN*, *H2AFY2*, *SMYD3*, and *DLGAP1*; pigmentation and lentigines, such as *BNC2*, *OCA2*, *SOD2*, *ARHGEF7*, and *PEX3*; and ECM remodeling and fibroblast identity, such as *MMP2*, *MMP9*, *HES1*, *KLF6*, *MFAP5*, *MGP*, *PDPN*, *PTGDS*, and *TGF-β1*. Collectively, these genes regulate collagen organization, elastin homeostasis, melanogenesis, oxidative-stress responses, and cellular senescence.

### 2.2. Differential Methylation Analyses-Dmp and Dmr

To determine whether the d3GHR genotype was associated with localized epigenetic differences, we compared the three genotype groups (fl/fl, fl/d3, and d3/d3) within the targeted CpG panel. Single-site differential methylation analysis was performed using linear models with empirical Bayes moderation and Benjamini–Hochberg false discovery rate (FDR) correction. Using predefined significance thresholds of FDR < 0.05 and |Δβ| ≥ 0.05, no differentially methylated probes (DMPs) were detected in any genotype contrast (fl/d3 vs. fl/fl, d3/d3 vs. fl/fl, or d3/d3 vs. fl/d3). These findings indicate that the d3GHR genotype was not associated with large, statistically robust single-CpG methylation differences within the targeted panel.

Because regulatory effects may occur at the regional rather than single-CpG level, we next evaluated differentially methylated regions (DMRs) using *bumphunter* with family-wise error rate (FWER) correction. Two promoter-proximal DMRs met the significance threshold of FWER < 0.05. The first DMR, located at chr15:74,726,847–74,726,961 and comprising eight CpG sites, was annotated near *CYP1A1* and showed hypermethylation in d3/d3 carriers compared with fl/d3 carriers (mean delta beta = +0.1575; *p* = 7.3 × 10^−5^; FWER = 0.014). The second DMR, located at chr6:159,761,152–159,761,592 and comprising four CpG sites, was annotated near *ACAT2* and showed hypermethylation in d3/d3 carriers compared with fl/fl participants (mean delta beta = +0.1652; *p* = 1.47 × 10^−4^; FWER = 0.026).

Thus, although no single-CpG effects were detected under stringent significance criteria, clustered promoter-level differences were observed. These regional differences may represent coordinated epigenetic signatures associated with the GHR exon 3 genotype. Of the two annotated genes, *CYP1A1* has previously been linked to pigmentation and photobiology, whereas Acetyl-CoA Acetyltransferase 2 (*ACAT2*) is primarily involved in lipid metabolism, particularly in the liver and intestine. This pattern suggests a potential systemic metabolic association rather than a direct skin-specific effect [11,12]. This interpretation is consistent with prior work showing that skin-based aging and tissue-damage markers, including skin fluorescence and collagen glycation, can integrate genetic, glycemic, and non-glycemic metabolic influences [2,3,4].

### 2.3. DNAm Age Clocks

DNA methylation (DNAm) clocks estimate biological age based on methylation levels at defined sets of CpG sites and enable comparison with chronological age. First-generation clocks include the pan-tissue Horvath clock, which is based on 353 CpGs, and the Hannum leukocyte clock, which is based on 71 CpGs. In contrast, second-generation clocks, such as PhenoAge, were developed to better capture health-related aging signals. Tissue-informed models, including the Skin & Blood clock, were designed to improve age estimation in specific tissues, including skin and hematologic samples [9].

In this study, age acceleration was calculated for each DNAm clock as the difference between DNAm age and chronological age (Δ = DNAm age − chronological age), with Δ > 0 indicating epigenetic age acceleration and Δ < 0 indicating epigenetic age deceleration. To further account for the influence of chronological age, we also calculated age-acceleration residuals (AARs) by regressing DNAm age on chronological age and analyzing the resulting residuals.

**Figure 1:**
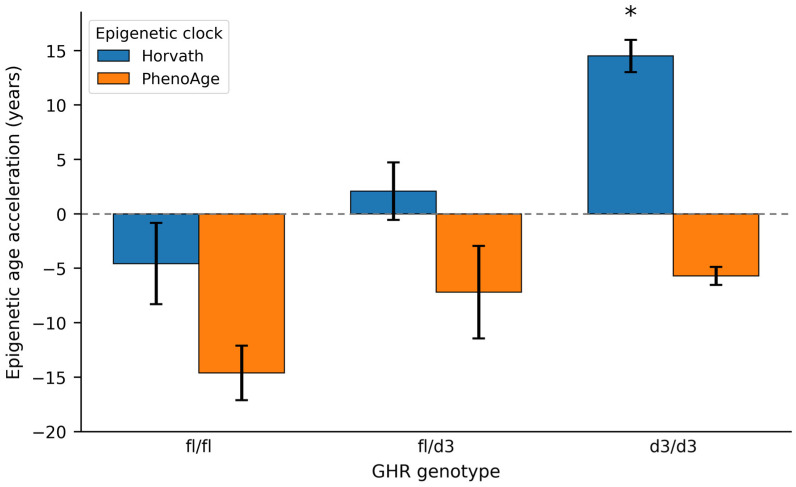
Age acceleration by GHR exon 3 genotype according to the Horvath and PhenoAge clocks. Mean age acceleration is shown across fl/fl, fl/d3, and d3/d3 genotype groups, calculated as DNAm age minus chronological age. The *x*-axis shows GHR exon 3 genotype, and the *y*-axis shows epigenetic age acceleration in years. Bars represent mean values, and error bars represent SEM. The dashed horizontal line indicates zero age acceleration. In this exploratory analysis, the Horvath clock showed nominally greater age acceleration in d3/d3 carriers compared with fl/fl participants (*p* = 0.0138, Tukey post hoc test). Given the very small d3/d3 subgroup, this finding should be interpreted as exploratory. * *p* < 0.05 vs. fl/fl (Horvath clock, Tukey post hoc test).

Across the three GHR genotype groups, only the Horvath DNAmAge clock showed a significant group effect for age acceleration (Δ; ANOVA F(2,18) = 5.08, *p* = 0.0179). Mean Δ values were −4.56 ± 9.86 years in fl/fl participants, +2.09 ± 8.75 years in fl/d3 participants, and +14.51 ± 2.57 years in d3/d3 participants, indicating greater epigenetic age relative to chronological age in d3/d3 carriers (Figure 1). Post hoc Tukey testing showed significantly greater age acceleration in d3/d3 carriers compared with fl/fl participants (mean difference ≈ 19.1 years, *p* = 0.0138), with a non-significant trend compared with fl/d3 participants (mean difference ≈ 12.4 years, *p* = 0.0998). The difference between fl/d3 and fl/fl participants was not statistically significant (*p* = 0.766).

Importantly, this signal persisted after adjustment for chronological age. Age-acceleration residuals differed significantly by genotype (ANOVA F(2,18) = 4.90, *p* = 0.0200). Post hoc Tukey testing again showed significantly greater AARs in d3/d3 carriers compared with fl/fl participants (*p* = 0.0157), with a non-significant trend compared with fl/d3 participants (*p* = 0.1147). The difference between fl/d3 and fl/fl participants remained non-significant (*p* = 0.2723).

In contrast, the Hannum and PhenoAge clocks showed no significant genotype differences for either raw age acceleration (Δ) or AARs (all *p* > 0.05). The Skin & Blood clock also showed no statistically significant genotype effect, although AARs exhibited a trend toward significance (ANOVA *p* ≈ 0.0558). Together, these findings suggest a clock-specific association rather than a uniform shift across DNA methylation-based aging measures.

## 3. Discussion

### 3.1. Clock-Specific Epigenetic Age Acceleration in D3ghr Carriers

d3GHR is a common variant associated with altered GH sensitivity and longevity-related phenotypes, including a reported male-specific longevity association [9]. In this study, homozygous d3/d3 carriers showed an exploratory signal of Horvath age acceleration according to the pan-tissue Horvath clock, with a mean age acceleration of +14.5 years. Notably, we found no significant single-CpG methylation differences in core structural skin-related genes but identified significant promoter-proximal DMRs near genes involved in metabolic and stress-response pathways, namely *ACAT2* and *CYP1A1*, each observed in distinct genotype contrasts.

The observation of Horvath age acceleration in d3/d3 carriers, despite previously reported longevity-related associations of d3GHR, should be interpreted cautiously. This pattern may reflect the known dependence of epigenetic clock outputs on tissue source, immune-cell composition, metabolic state, and clock design. In addition, because this cohort was not enriched for individuals of very advanced age or longevity-range individuals, the observed Horvath signal should not be interpreted as direct evidence against a longevity-associated phenotype. Prior studies suggest that d3GHR may be linked to altered GH/IGF-1 signaling and enhanced cellular resilience [9,13,14]. Therefore, the Horvath signal observed here may reflect systemic endocrine, metabolic, or immune features captured in whole blood, rather than accelerated structural aging of skin-related molecular programs.

### 3.2. Divergence Between Chronological and Biological Clocks

The discrepancy between the first-generation Horvath clock and second-generation clocks such as PhenoAge highlights the importance of clock selection when interpreting epigenetic age. Horvath-based measures may be influenced by metabolic state and immune-cell composition, both of which can shift in response to altered GH signaling [15,16]. For example, differences in naive-to-memory T-cell ratios in d3GHR carriers could generate an immunophenotype interpreted by the clock as “older,” reflecting systemic adaptation rather than true biological decline.

Consistent with this interpretation, PhenoAge, which is calibrated to physiological dysregulation and mortality risk, showed a trend toward epigenetic deceleration, aligning more closely with the reported longevity-related phenotype [16]. This divergence underscores the need for multi-clock approaches and tissue-specific measures when assessing biological aging [17]. In addition, baseline differences in functional status, including lower physical activity and performance scores among d3/d3 carriers, may have further influenced immune–metabolic profiles and first-generation clock readouts in this cross-sectional setting. However, despite the older age and lower functional status of d3/d3 carriers, the absence of consistent acceleration across second-generation or tissue-informed clocks may suggest genotype-related resilience rather than uniform epigenetic decline.

### 3.3. Genotype-Linked Methylation Signatures Near Acat2 and Cyp1a1

Two promoter-proximal DMRs provide potential mechanistic insight (Table 3): a CYP1A1-associated region showing hypermethylation in d3/d3 carriers relative to fl/d3 carriers, and an ACAT2-associated region showing hypermethylation in d3/d3 carriers relative to fl/fl participants.

The DMR near *ACAT2* may reflect epigenetic modulation of metabolic pathways, particularly cholesterol biosynthesis and systemic energy metabolism [18]. Experimental mouse models suggest that *ACAT2* overexpression can promote higher energy expenditure and bile acid biosynthesis. Although DNA methylation does not always correlate linearly with gene expression, promoter-adjacent DMRs may be associated with altered transcriptional activity. We therefore hypothesize that the *ACAT2*-associated DMR observed in d3/d3 carriers may reflect genotype-linked remodeling of metabolic flux. Such remodeling could generate metabolic signals that a broad pan-tissue clock, such as the Horvath clock, may interpret as accelerated epigenetic aging, without necessarily indicating structural decline in skin-related pathways [15].

The second DMR, located near *CYP1A1*, may reflect pathways related to detoxification, oxidative stress, and photobiology. Hypermethylation in this region may represent an adaptive stress-response signature, consistent with enhanced cellular resilience previously reported in d3GHR-related models [14]. Together, these findings suggest that the Horvath age-acceleration signal observed in d3/d3 carriers may capture metabolic and immunological adaptation rather than structural senescence.

### 3.4. Strengths and Limitations

A key strength of this study is the identification of genotype-linked epigenetic signatures in a non-Druze population, extending prior work conducted in genetically homogeneous and consanguineous Druze cohorts [9]. In that previously published Druze d3GHR cohort, d3GHR genotype was associated with distinct DNA methylation patterns, including significant DMPs and DMRs, and d3/d3 carriers showed a favorable DNAmFitAge profile relative to WT and heterozygous individuals [9]. These findings provide supportive external context for a genotype-linked epigenetic phenotype. However, because that cohort differed from the present study in ancestry, family structure, age distribution, and analytical framework, these findings should be considered supportive external evidence rather than a direct replication of the present DMR and clock findings.

Several limitations should be acknowledged. The principal limitation is the very small number of d3/d3 homozygotes, which included only three individuals. This subgroup size is insufficient for definitive, reproducible genotype-specific inference, reliable estimation of within-group variance, or robust assessment of distributional assumptions. Therefore, all findings involving d3/d3 carriers, including the Horvath age-acceleration and DMR signals, should be interpreted strictly as exploratory and hypothesis-generating rather than confirmatory.

Nevertheless, because d3/d3 homozygosity represents a minority genotype subgroup, small numbers are expected in exploratory cohorts based on available samples. Moreover, the observed direction of epigenetic variation is broadly consistent with prior d3GHR-related methylation, GH/IGF-1 signaling, stress-resilience, metabolic adaptation, and longevity-associated findings. These observations may therefore be useful for designing larger validation studies, but they should not be viewed as definitive evidence of a reproducible d3/d3-specific epigenetic phenotype.

In addition, we detected no single-CpG differential methylation in a targeted panel of core structural and remodeling genes, including *COL1A1*, *COL1A2*, *ELN*, and *MMP2/9*. Although this finding suggests that the d3GHR variant is not associated with robust alterations in dermal-structure gene programs in circulating cells, blood-derived methylation remains only an indirect proxy for skin biology. While blood-based epigenetic markers may correlate with facial-aging signatures [17], definitive assessment of structural preservation requires tissue-specific evaluation in skin-derived samples. An additional limitation is the absence of direct clinical or physiological measures of skin aging, such as validated wrinkle grading, skin elasticity, dermal thickness, imaging-based skin age assessment, or skin-derived tissue methylation. Therefore, the present findings should not be interpreted as evidence of altered clinical skin aging in d3GHR carriers. Rather, they reflect blood-based epigenetic signatures in aging-related and skin-aging-related pathways that require tissue-specific validation.

### 3.5. Future Directions

Future studies should examine DNA methylation directly in skin-derived cells, particularly fibroblasts and keratinocytes, ideally incorporating skin-specific epigenetic clocks such as VisAgeX [19]. Larger cohorts integrating DNA methylation, gene expression, and metabolic profiling, including bile acid measurements, will be necessary to validate the proposed mechanisms [18]. Establishing a causal pathway linking d3GHR genotype, *ACAT2* modulation, and metabolic remodeling will require integrated multi-omics approaches.

## 4. Materials and Methods

### 4.1. Study Population and DNA Extraction

We recruited 21 unrelated adults aged 39–70 years, including 13 females and 8 males, from Laniado Medical Center, Israel. This was an exploratory pilot study based on available samples. The age range of 39–70 years reflected the available cohort after sample availability and quality-control filtering. No formal a priori power calculation was performed. Future studies should include larger cohorts with broader age distributions to improve age-acceleration modeling and genotype-based inference. Given the small overall sample size and the very small d3/d3 subgroup (*n* = 3), the study was underpowered for definitive genotype-based inference, and all genotype-based analyses should be interpreted as exploratory and hypothesis-generating.

All study procedures were conducted in accordance with the Declaration of Helsinki and were approved by the local Institutional Review Board (0150-16-LND). Written informed consent was obtained from all participants.

Whole blood samples (9 mL) were collected in EDTA tubes. Genomic DNA was extracted using the FlexiGene DNA Kit (Qiagen GmbH, Hilden, Germany) and quantified with a NanoDrop One/OneC Microvolume UV-Vis spectrophotometer (Thermo Fisher Scientific, Waltham, MA, USA).

### 4.2. Ghr Exon 3 Genotyping

Genotypes for the *GHR* exon 3 deletion were determined using a multiplex PCR assay designed to distinguish the deletion allele (d3, 532 bp) from the full-length allele (fl, 935 bp). The 20 μL reaction mixture contained Taq 2× Master Mix and template DNA. Thermal cycling consisted of an initial denaturation step at 94 °C for 5 min, followed by 35 cycles of denaturation at 94 °C for 30 s, annealing at 60 °C for 30 s, and extension at 72 °C for 90 s, with a final extension at 72 °C for 7 min.

PCR products were separated by size on a 1.5% ethidium bromide-stained agarose gel. To confirm the deletion, the 935 bp and 532 bp bands were excised and purified using the GeneJET Gel Extraction Kit (Thermo Fisher Scientific, Waltham, MA, USA; Cat. No. K0692), followed by Sanger sequencing performed by Macrogen sequencing services.

### 4.3. Methylation Profiling and Bioinformatics

Genome-wide DNA methylation was assessed using the Infinium MethylationEPIC v2.0 BeadChip (Illumina Inc., San Diego, CA, USA). Raw IDAT files were processed in R (v4.3.2; R Core Team, Vienna, Austria) using the *minfi* (v1.48.0). Background and dye-bias correction were performed using *preprocessNoob*, followed by SWAN normalization.

Quality control included removal of samples with call rates below 96.5% and filtering of probes with poor detection *p* values, probes containing known SNPs, and probes with known mapping issues. As a secondary exploratory analysis, we analyzed a targeted subset of 1098 CpG sites mapped to genes involved in wrinkling, dermal elasticity, pigmentation, and extracellular matrix remodeling [1,6,7,8,20].

### 4.4. Differential Methylation Analysis

Single-site differential methylation was evaluated using linear models with empirical Bayes moderation implemented in the *limma* (v3.58.1) [21]. Genotypes were included as the factor of interest, and *p*-values were adjusted for multiple testing using the FDR procedure. Significant DMPs were defined using predefined thresholds of FDR < 0.05 and |Δβ| ≥ 0.05.

For regional analysis, DMRs were identified using *bumphunter* (v1.44.0) applied to smoothed methylation values. Regions were required to contain at least four CpG sites and to meet a Δβ cutoff of 0.05. Statistical significance for DMRs was defined as a FWER < 0.05, estimated using 1000 permutations. All identified regions were annotated to the hg38 reference genome and characterized according to gene proximity, genomic feature, and CpG context.

### 4.5. Epigenetic Clock and DNA Methylation (DNAm) Age Calculation

Biological age was estimated using the Horvath, Hannum, PhenoAge, and Skin & Blood DNA methylation clocks. Age acceleration was quantified using two metrics: the raw difference between DNAm age and chronological age, calculated as DNAm age − chronological age, and AARs, obtained by regressing DNAm age on chronological age and analyzing the resulting residuals.

### 4.6. Statistical Analysis of Age Acceleration

Group differences in DNAm age-acceleration metrics across genotypes were evaluated using one-way ANOVA. Given the small overall sample size and the very small d3/d3 subgroup (*n* = 3), all genotype-based statistical comparisons were interpreted as exploratory. Model assumptions were assessed using the Shapiro–Wilk test for normality and Levene’s test for homogeneity of variance; however, these tests were considered limited in reliability because of the small subgroup sizes. Significant group effects were followed by post hoc pairwise comparisons using Tukey’s honestly significant difference test or Welch’s t tests with Holm correction, as appropriate. Kruskal–Wallis tests were performed as non-parametric sensitivity analyses.

Given the exploratory nature of the study, no formal a priori power calculation was performed. Given the present sample size, particularly the d3/d3 subgroup of three participants, the study was underpowered for definitive genotype-based inference. The analyses were therefore intended to identify preliminary signals for future validation rather than to provide confirmatory statistical evidence.

All analyses were conducted in R (v4.3.2; R Core Team, Vienna, Austria) and Python (v3.11.5; Python Software Foundation, https://www.python.org, accessed on 1 June 2026), with a two-sided significance threshold of α = 0.05.

## 5. Conclusions

The d3GHR genotype was associated with a distinct blood-based epigenetic profile characterized by promoter-proximal methylation differences near genes involved in metabolic and stress-response pathways. We found no strong evidence of differential methylation in core structural skin-aging genes, suggesting that the observed signatures may reflect systemic metabolic adaptation rather than direct remodeling of skin-structure programs.

Because only three d3/d3 homozygotes were included, these findings cannot establish definitive or reproducible d3/d3-specific epigenetic effects. Rather, they identify a biologically plausible direction of association, consistent with prior d3GHR-related studies, that should be tested in larger independent cohorts using comparable methylation and clock-analysis frameworks. In addition, because no direct clinical or physiological measures of skin aging were available, the present results should be interpreted as blood-based epigenetic aging findings rather than evidence of altered clinical skin aging.

## Figures and Tables

**Table 1:** Demographic characteristics and chronological age distribution by GHR exon 3 genotype.

Genotype	N	Age Range (Years)	Mean Age (Years)	Median Age (Years)	Age IQR (Years)	Gender (Female/Male)
fl/fl	7	50–69	57.7	55	53.0–62.0	6 F/1 M
fl/d3	11	39–67	54.7	57	50.5–59.0	5 F/6 M
d3/d3	3	55–70	64.7	69	62.0–69.5	2 F/1 M
Total	21	39–70	57.1	57	51.0–64.0	13 F/8 M

**Table 2:** Functional status measures by GHR exon 3 genotype.

Genotype	Physical Activity (Phisact; 1–3), Mean ± SD	Physical Performance Score (0–8), Mean ± SD
fl/fl	1.71 ± 0.76	4.71 ± 1.11
fl/d3	1.73 ± 0.79	4.00 ± 3.03
d3/d3	1.00 ± 0.00	3.67 ± 1.15
Total	1.62 ± 0.74	4.19 ± 2.29

phisact = physical activity score. Physical performance score was derived from standardized functional assessments, including gait speed, grip strength, balance, chair-stand performance, and step endurance, with higher scores indicating better physical performance [10].

**Table 3:** Top differentially methylated regions associated with GHR exon 3 genotype. This table summarizes promoter-proximal differentially methylated regions (DMRs) associated with GHR exon 3 genotype across the tested contrasts.

Region	Contrast	CpGs	Gene	Mean Δβ	Direction	*p* Value	FWER	Impact
chr6:159761152-159761592	d3/d3 vs. fl/fl	4	ACAT2	+0.1652	Hyper	1.47 × 10^−4^	0.026	Fatty acid metabolism; energy expenditure
chr15:74726847-74726961	d3/d3 vs. fl/d3	8	CYP1A1	+0.1575	Hyper	7.35 × 10^−5^	0.014	Oxidative stress; photobiology

Δβ is presented from the perspective of d3/d3 relative to the comparator genotype. Positive values indicate higher methylation in d3/d3. Hyper = hypermethylated. FWER = family-wise error rate.

## Data Availability

The data presented in this study are available on request from the corresponding author. The data are not publicly available due to privacy and ethical restrictions related to human genomic data and the terms of the Institutional Review Board approval.

## Abbreviations

The following abbreviations are used in this manuscript:

AAR	Age-Acceleration Residual
ACAT2	Acetyl-CoA Acetyltransferase 2
CpG	Cytosine-phosphate-Guanine
CYP1A1	Cytochrome P450 Family 1 Subfamily A Member 1
d3GHR	Exon-3 deleted Growth Hormone Receptor
DMP	Differentially Methylated Probe
DMR	Differentially Methylated Region
DNAm	DNA Methylation
ECM	Extracellular Matrix
FDR	False Discovery Rate
flGHR	Full-length Growth Hormone Receptor
FWER	Family-Wise Error Rate
GH	Growth Hormone
GHR	Growth Hormone Receptor
IGF-1	Insulin-like Growth Factor 1
